# Psychotherapy Research Domain Criteria: functional mechanisms of treatment and a basic theory of the mind

**DOI:** 10.3389/fpsyt.2025.1549976

**Published:** 2025-06-19

**Authors:** Nico Rohlfing, Martin Pum, Udo Bonnet, Katja Koelkebeck, Norbert Scherbaum

**Affiliations:** ^1^ Department of Addictive Behaviour and Addiction Medicine, LVR-Hospital Essen, University of Duisburg-Essen, Essen, Germany; ^2^ Department of Psychiatry and Psychotherapy, LVR-Hospital Essen, University of Duisburg-Essen, Essen, Germany; ^3^ AVS Social and Health Center, Klagenfurt, Austria; ^4^ Department of Psychiatry, Psychotherapy, and Psychosomatic Medicine, Evangelisches Krankenhaus Castrop-Rauxel, Academic Teaching Hospital of the University of Duisburg-Essen, Essen, Germany; ^5^ Center for Translational Neuro- and Behavioural Sciences (CTNBS), University Hospital Essen, Essen, Germany

**Keywords:** Research Domain Criteria, principles of change, needs, cognitive bias, reappraisal

## Abstract

The Research Domain Criteria (RDoC) approach is a tipping point in psychotherapy and introduces a new development in the treatment of mental disorders. The linking of clinical syndromes with their biological foundation shifts the emphasis of research and methodology on biology and increases the falsifiability of therapy schools, trends, and paradigms in psychotherapy. Interventions are not exclusively assessed according to their efficacy anymore; they focus on biological mechanisms and aim to alter them in an evidence-based way. At the same time, research benefits from the clinical expertise of experienced practitioners and proven treatment concepts. With this heterogeneity and with the decline of diagnosis-specific treatment, a vacuum occurs with respect to a basic theory on the functionality of the mind and the central approach for treatment. The mind can be assessed precisely by biologically based functional mechanisms. Needs could be moved into the center of treatment and their neural mechanisms, which overlap with addiction and reward processing, are the interface between universally valid or nomothetic processes and an individualized idiographic treatment. The RDoC approach will prospectively lead to a huge integration of proven treatment concepts to develop innovative evidence-based interventions and a basic theory of the mind in the sense of a universally valid neuropsychotherapy. The rationale was to define a central approach to and a RDoC perspective on psychotherapy.

## Introduction

The US National Institute of Mental Health has developed the Research Domain Criteria (RDoC) approach to explore the underlying biological causes of mental disorders and has established a research framework to link and integrate current clinical syndromes with basic biological and behavioral components (1). The current version of the RDoC framework consists of six domains of human functioning (2). The domains represent contemporary knowledge about major systems of emotion, cognition, motivation, and social behavior (2). The goal seeks to understand mental functioning in continuous valid dimensions ranging from functional to pathological.

In contrast, the diagnostic systems in psychiatry, the International Classification of Diseases (ICD) and the Diagnostic and Statistical Manual of Mental Disorders (DSM), are based on a categorical approach (3). They define symptoms, specify symptom clusters, and thus offer a standardized categorization of mental diseases. On the one hand, these systems have provided a standardization and thus a common language for mental diseases across the world (3). On the other hand, the high comorbidity, clinical heterogeneity, and exclusion of biomarkers are significant limitations of the present diagnostic systems in psychiatry. In any case, this categorical descriptive approach, with its limitations, calls into question the validity of current symptom clusters and diagnoses.

The domains and subordinated constructs of the RDoC framework represent biopsychological processes and mechanisms and they are regarded as a continuum between the functional and pathological (4). In this sense, RDoC rethinks psychopathology by turning away from current descriptive symptom clusters to a new biological and functional transdiagnostic psychopathology (5). The subordinated constructs specify the respective domain (2). The constructs are assessed in units of analysis encompassing the entire spectrum of methods from genes, circuits, observed behaviors, self-reports, and paradigms. The reason for the broad range of methods is to promote multi-level analysis and to cover and integrate all relevant disciplines from psychology, via neuroscience, to biology (4, 6). The dimensions and constructs are not considered as final or static, but as a work in progress or dynamic (7). They are constantly adapted to and extended by the current research status.

However, this has not yet led to an integration of the disciplines into a basic causal model of psychopathology (5). New findings in neuroscience do not align with a specific diagnosis, and a particular symptom may be applicable to various diagnoses. Thus, categorical diagnoses may not be suitable independent variables for research, because they just do not represent homogenous groups (8). The heterogeneity of psychotherapeutic methods has mainly been caused by the level of contemporary knowledge in the past (9). The missing validity of the current diagnostic system results in manualized treatments for specific diagnoses, which are based on invalid assumptions (10). In clinical psychology, integrative considerations exist that psychotherapy has common principles of change regardless of therapy schools (9). However, it is impossible to grasp these principles of change precisely with traditional psychotherapy research methodology. Due to psychotherapy research methodology, upcoming therapy approaches can be highly promoted and their innovation level is often exaggerated (9). It has therefore been the inevitable next step to extend psychotherapy research with evidence-based medicine and the investigation of neural correlates of mental processes. In the future, interventions will increasingly be linked with biological mechanisms and aim to alter them, which provides a completely new path and measurement to evaluate interventions (5). This change marks a tipping point in psychotherapy, increases the falsifiability of therapy schools, and introduces a new development in the treatment of mental disorders, shifting psychotherapy towards intervention science (11). Nevertheless, it lacks a foundation for the exchange between practice and basic research as well as a guideline for therapeutic practice.

The neuropsychotherapy approach already formulates an integrative model of psychological functioning, which accounts for the proceedings, processes, and mechanisms in therapy (12). In comparison, the dynamic RDoC approach enables a more profound, biological understanding of mental disorders. Psychotherapy can be regarded as the accurate evaluation of experiences, which occurs within the processes and underlying neural mechanisms of prediction errors and adaptive expectations and comprises goal attainment and need satisfaction (13). As the individual needs of a patient are the basis for every treatment, needs naturally form the most appropriate central approach to treatment and their neural mechanisms are the interface between universally valid or nomothetic processes and an individualized idiographic treatment (10). From a phylogenetic or evolutionary perspective, the reliving of past events in the “here-and-now” enables us to draw on mental representations, which allow for goal achievement (14). Re-experiencing could thus be the most fundamental functional mechanism of the mind, increasing the predisposition for mental diseases at the same time. A core principle of treatment in psychotherapeutic approaches is the reappraisal or restructuring of semantic representations (15). Semantic representations determine cognitive control and behavior (15–17). Appraisals are generalized regularities of experience, whereby the regularities are neuronally disconnected from the related experiences. Autobiographical memory maintains a coherent sense of self over time (18). The key feature of semantic representations and neural processing is, therefore, self-containing biases. At the same time, behavior is automated unconsciously based on contingency and then does not require voluntary attention, but is goal-directed. Psychotherapy extends appraisal, makes clients aware of automated behavior and creates a functional behavioral reaction, in which the implicit corresponds with explicit emotion regulation, and which establishes cognitive control over emotion regulation and self-regulation.

In a basic theory of the mind, needs could form a central approach to treatment, re-experiencing could be a fundamental, evolutionarily justified functionality, and psychotherapy can be regarded as the accurate evaluation of experiences with reappraisal interventions. Self-containing biases represent a crucial target point for interventions. This narrative review outlines psychotherapy from a RDoC perspective with needs as the central approach and variable, evaluation of experiences as the main mechanism, biases as the main target, and reappraisal as the main intervention of psychotherapy. The rationale was to define a central approach to treatment, a fundamental functionality of the mind, and the process of psychotherapy in order to overcome heterogeneity and link and integrate different fields into an RDoC perspective on psychotherapy.

## Psychotherapy research methodology, principles of change and intervention science

Even at the very beginning of the field of psychotherapy, Freud´s followers began to diverge and develop their own individual approaches to explain how people change (9). Since then, a tendency toward proliferation in different theoretical approaches to psychotherapy has developed continuously (9). The institutionalization of therapy schools with social, political, and economic contact points may have promoted competition, separation, and tenacity among different paradigms. Notwithstanding these framework conditions, different schools of thought have simply been unable to unify their models and there is neither a set of unified techniques or interventions, nor a specific theory, core of knowledge or consensus about psychotherapy (9). This has led to the existence of approximately 500 different schools of thought, with the majority of clinicians stating that they would follow more than one approach in their clinical work (19, 20).

The initial practice of psychotherapy was solely based on clinical observation and experience (9). In its first phase, between the 20s and 50s of the past century, psychotherapy research started with the question of whether treatment had an outcome at all and if so, to what extent (21). Outcome research investigates the effectiveness of therapy under real conditions or the efficacy under ideal conditions, and efficacy studies are conducted in randomized control trials (22). The following process and process-outcome research deals with the study of the processes in therapy, their associations with the outcome, and the specific effect of these processes (21). Meta-analyses are being used to calculate the effect size for a particular intervention. This traditional psychotherapy research has so far not been able to fully grasp mental functioning with its methods in any of its past phases. Additionally, there is a gap between this research field and practice due to considerable reservations of some clinicians whose valuable expertise unfortunately becomes lost in the scientific debate (9). A challenge for the proclaimed effect factors of different therapy schools and for mental processes in general is the abstraction level and the involved difficulties in operationalizing them in experiments. For this reason, and due to psychotherapy research methodology, it may be possible to highly promote upcoming therapy approaches and exaggerate their innovation level within the field of psychotherapy research (9).

Beyond the proliferation of heterogeneity, integrative considerations and efforts also exist in clinical psychology, which correspond with the idea of the RDoC approach (9). These considerations are that psychotherapy has common principles of change regardless of certain therapy schools, specific interventions, or techniques. The principles of change comprise the clients´ therapy expectation, motivation, and problem awareness; the therapeutic alliance; and the promotion of corrective experiences and reality testing. These principles are considered crucial for the outcome of every treatment. It was suggested that it is better to shift the research focus from the efficaciousness of a school of therapy in treating a DSM disorder to these transtheoretical principles (23). Databased or empirically supported principles of change will advance progress more than therapy school treatments or manuals (23, 24). The principles of change confirm the significance of clinical observation and converging methods to obtain evidence for reliable conclusions about psychotherapy, which will reduce separation and promote exchange among different paradigms in clinical practice (9).

Thus far, specific therapy protocols have mainly targeted latent disease entities and interventions have been evaluated on the basis of their efficacy (19, 20). In the RDoC approach, interventions will increasingly be linked with biological mechanisms and aim to alter them, which provides a completely new path and measurement to evaluate interventions (5). There are two conceivable starting points for the implementation of RDoC into existing psychotherapy and for the design of evidence-based interventions. One starting point is in psychopathology, collecting and including multi-level data across multi-domains to identify clusters or biotypes first as a basis for the subsequent design of interventions (19). The second starting point is the selection of RDoC constructs for interventions with high functional relevance to a disorder or cluster of disorders to test whether they are mechanisms of change and promote efficacy (19).

For example, Clementz and colleagues were able to identify three biotypes of psychosis (25). They collected brain function biomarkers in individuals with schizophrenia, schizoaffective disorder, and bipolar disorder with psychosis; individuals` relatives; and control subjects. The resulting three neurobiologically distinct psychosis biotypes did not correspond with clinical diagnosis boundaries and provided new biologically differentiated approaches for interventions. Van Dam and colleagues reported behavioral and biological dimensional measures for mental dysfunction and for mental function (26). Notably, and in line with the RDoC approach, measures of mental health and functionality were also detected (26). Just as for the distinct psychotic biotypes, the measures also captured variations beyond contemporary diagnostic categories. Thus, the advantage of this data-driven starting point is the opportunity to open up completely new fields beyond current diagnostics for the subsequent design of interventions.

Training for Awareness, Resilience and Action (TARA), for example, is a novel group programme for adolescent depression (27, 28). Blom and colleagues used developmental neurobiological evidence on depression as a guideline for the design of TARA and aligned it with the RDoC. Due to limited top-down cognitive control, the programme prefers and promotes bottom-up strategies such as breathing exercises to increase vagal afference and improve autonomic regulation (27). Relevant RDoCs were identified, and sustained threat, loss, social processes, and reward learning were prioritized as target constructs. At the same time, interventions have been selected from proven modern psychotherapy techniques and ordered in line with neurobiological evidence and efficacy. The authors disentangled RDoC and existing therapy concepts and interlinked them skillfully and most effectively for TARA. A pilot study produced validity evidence for the predicted target constructs, including anxiety symptoms, and for the efficacy of TARA (23).

Generally, a challenge for mental processes is the abstraction level and the difficulties involved in operationalizing them in experiments. Psychotherapy research methodology has not been able to grasp mental functioning (9). Thus, it has been possible to exaggerate the innovation level of upcoming therapy approaches and promote them within the field of psychotherapy research (9). With the introduction of the RDoC, biological mechanisms have come to the forefront in diagnosis and research and the gain in knowledge in this field is enormous (29). This change introduces a new development in the treatment of mental disorders, increases the falsifiability of therapy schools, and shifts psychotherapy towards intervention science (11). In contrast to the concepts and assumptions of certain therapy schools, these mechanisms are not theoretical, but biological and thus falsifiable and ultimately empirical. The transtheoretical principles of change are also not limited to certain therapy schools and illustrate the significance of clinical expertise and observation. As they are universally reported from everyday clinical practice, they most likely have a biological foundation and can thus be considered as part of those mechanisms. The RDoC approach, therefore, only enables a general understanding of mental disorders. The missing consideration of the biological foundation of mental processes and the level of contemporary knowledge in the past are probably the major factors for the lack of a basic model of the mind (9).

## Needs in the center of treatment

Neglect of patients´ needs and a preference for neurobiological approaches have been regarded as deficiencies of the RDoC (29). However, this conception seems questionable. From an epistemological perspective, it is no matter of discretion and hard to imagine that any mental process has no biological origin. This may even be true for individual characteristics such as subjective reward processing (30). For example, in a pilot study, subjective reward processing was assessed in abstinent cannabis users with the monetary incentive delay task and subjective value was not detached from reward parameters, but was modulated from expectancy and reward by the insula. The underlying neural mechanisms are a fundamental target point for treatments, interventions, and cognitive behavioral therapy (30). Therefore, a (neuro-) biological approach cannot be seen as a preference or deficiency of the RDoC, but is the indispensable prerequisite for a general understanding and the development of a basic theory of the mind. In addition, the findings for subjective reward processing show that even more abstract and individual characteristics, such as needs, can be traced back to an underlying neural mechanism and a systematic process. The investigation of neuronal mechanisms, neurobiological markers, and the systematic processes of individual characteristics could be a future direction for psychotherapy research. However, a huge challenge is to implement these findings into clinical interventions or diagnostics so that patients can benefit in a routine therapeutic setting.

In psychotherapy, in the current diagnostic systems, treatment protocols have been built for specific diagnoses, and this manualized approach has been the dominant paradigm for half a century (10). The diagnoses are exclusively defined by the current diagnostic systems, yet they are hypothesized, unproven latent disease entities (10). Therefore, the starting point or the basic assumption of a specific manualized treatment is already invalid. Some of the previously mentioned aspects may be consequences of this flaw and provide further indirect evidence for the missing validity of the diagnoses. For example, if a manualized treatment was highly specific and effective, then a particular disease could be treated exclusively with this protocol. In this case, the existence of common transtheoretical and -diagnostic mechanisms of change was unlikely or even impossible. Accordingly, the invalid diagnostic access hampers a correct understanding of the mind, because manualized treatments are built on invalid basic assumptions and do not take transdiagnostic common mechanisms of the mind into account. This distorted approach inhibits the progress of psychotherapy. Hayes and colleagues reason that manualized, syndrome-specific psychotherapy has failed to achieve conceptual and treatment utility (10). The ICD and DSM provide a standardization and common language for mental disease, though merely in the sense of the lowest common denominator. The standardization gives some guidance to both the therapist and the patient in the treatment, but at the same time promotes an unconscious disproportionate diagnostic focus and by that a pathologizing perspective of therapists and patients (31). However, it would be diagnostically difficult to find a distinguishing criterion for mental diseases on the level of needs.

Hayes and colleagues propose a process-based approach for the understanding and treatment of the mind (10). The focus of process-based therapy is on empirical biopsychosocial processes of change that are important to long-term goals and outcomes. As an alternative to contemporary psychiatric nosological systems, they provide an evolutionary meta-model to consider and accommodate any set of evidence-based change processes. In the near future, the capture of these processes would enable an idiographic psychotherapy: that is, an individualized functional process could be selected for a person’s specific psychological problem in their unique current circumstances. In contrast to the nomothetic, protocolized treatments, individualized treatments would utilize case formulation and functional analysis that fit the needs of given individuals based on known processes of change (10). For example, humanistic therapy also assumes an individualized treatment with a focus on the person´s unique history and maladaptive adjustment strategies (10). However, this qualitative approach still lacks experimental methods to produce a systematic and proven classification and intervention system (10). An idiographic, process-based approach was criticized for lacking evidence of its superiority over a nomothetic, manualized treatment (31). The high significance of the therapeutic alliance for the outcome of therapy, even in comparison with interventions, indicates the meaningfulness of an individualized proceeding and the idiographic approach (32). According to Hayes and colleagues, the key question for individually tailored interventions and even the future of evidence-based care is “*What core biopsychosocial processes should be targeted with this client given this goal in this situation, and how can they most efficiently and effectively be changed*?” (10).

A simple answer could possibly already be provided to this essential question: the processes and neural mechanisms of needs in the sense of basic psychological needs (12). The clarification of motivation and goal-directed behavior is a clinically significant, superordinate, transtheoretical principle of change (29). On the one hand, the individual needs of a patient serve as the basis for every treatment and therefore, needs naturally form the most appropriate central approach to treatment. On the other hand, their mechanisms and neural correlates, such as reward processing, are the interface between a universally valid or nomothetic process and an individualized idiographic treatment (12, 30).

**Figure 1:**
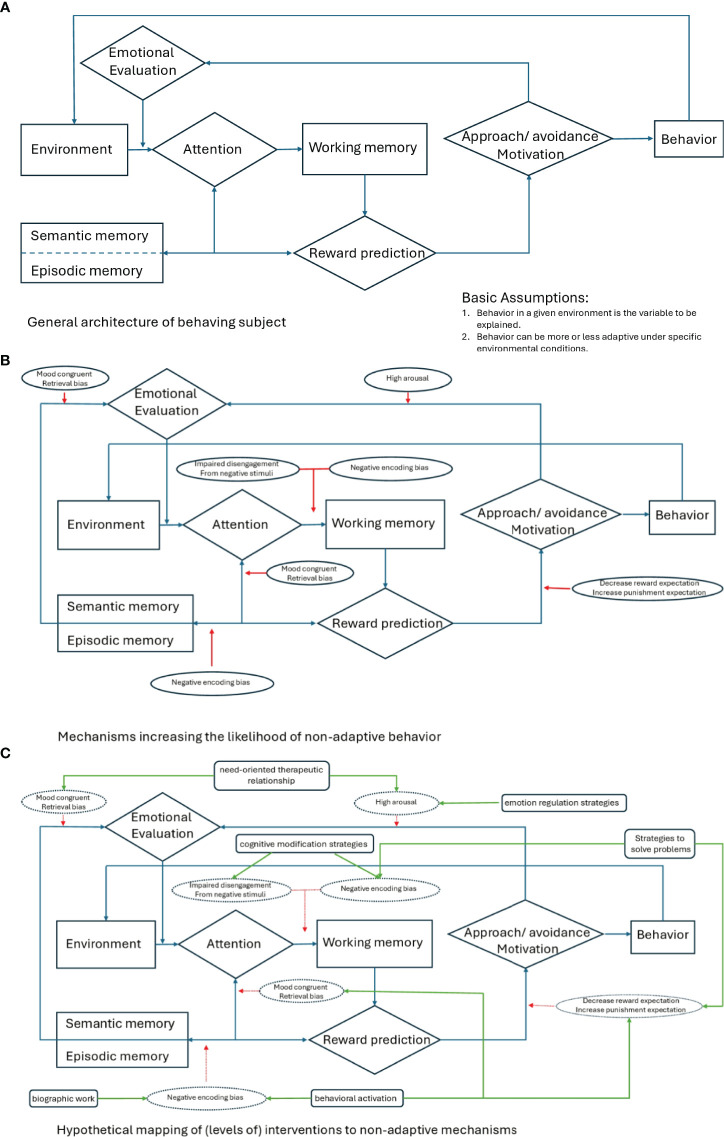
General architecture of the subject’s behavior **(a)**, mechanisms of non-adaptive behavior **(b)**, and interventions to non-adaptive mechanisms **(c)**.

Needs already build the central approach to treatment in neuropsychotherapy and serve as its foundation (12). According to Grawe, *“the goals a person forms during his or her life ultimately serve the satisfaction of distinct basic needs”* (12). Neuropsychotherapy links therapy with neuroscience and provides a multidisciplinary meta-framework for the therapeutic alliance, techniques and processes, and the underlying neural mechanisms (33). Consistency is considered the ultimate basic principle of mental functioning and is directly related to needs. Individual goals are traced back to the four key psychological needs of attachment, control/orientation, pleasure/avoidance of pain, and self-enhancement. These evolutionary-sounding basic needs elicit a cortical-driven approach or limbic-driven avoidance via implicit motivational schemata, which aim at goal attainment and need satisfaction or consistency. With needs in the center and these related concepts, neuropsychotherapy formulates an integrative model of psychological functioning, which accounts for the etiology and maintenance of mental disorders and the therapeutic stance, proceeding, processes, and mechanisms in therapy (12). As for clinical application and practical steps, need satisfaction, consistency, and goal attainment have the highest priority in the arrangement of the therapeutic alliance and the therapeutic stance focuses on the neural underpinnings of behavior (33). This stance promotes empathy, reduces patients´ self-blame, and eliminates stigma in contrast to a pathologizing perspective. It reduces symptom stress, gives patients access to their resources and self, and facilitates the approach. Such a therapeutic alliance and stance enhance self-effectiveness and finally promote positive need-satisfying experiences and positive social interactions in line with patients´ goals (33). The therapeutic stance in neuropsychotherapy is an example of an application of needs in a clinical setting (**Table 1**). The main outcome measures are need satisfaction, goal attainment, and the quality of the therapeutic alliance, and they target mood-congruent retrieval bias and high arousal (**Figure 1c**). From a methodological perspective, plan analysis is a case conceptualization instrument in psychotherapy, in which a person’s behavior is placed in relation to their needs. It is based on the assumption that behaviors are repeated and consolidated into implicit structures of action organized to serve a specific purpose and can be applied irrespective of therapy schools (34). The Questionnaire for the Analysis of Motivational Schemas (FAMOS) measures the motivational goals of psychotherapy patients and can be used as an assessment tool for case formulations and for change in psychotherapy (35). The Basic Psychological Need Satisfaction Scales is a set of original questionnaires that assess the degree to which people feel satisfaction in the three needs of competence, autonomy, and relatedness (36, 37). The neuropsychotherapy approach focuses on the proceedings and processes of psychotherapy (12).

**Table 1:** Needs as RDoC-variable and in the context of psychotherapy, clinical application and research.

	Psychotherapy	Clinical application	Addiction research	RDoC-variable
**Needs**	Need satisfaction	Therapeutic stance	Needs → goals	Cognitive control (Construct)
	Goal attainment			Goal selection (Subconstruct)

In comparison, the dynamic RDoC approach enables a more profound, biological understanding of mental disorders. In the RDoC approach, needs can be ascribed to the construct of cognitive control of the domain cognitive systems. Cognitive control modulates the operation of cognition and emotion for goal-attainment and need satisfaction (2). It consists of the subconstructs goal selection and updating. Goal selection refers to the cognitive process of choosing among potential outcomes, actions, or behaviors. Goal updating involves refreshing the cognitive content related to specific potential outcomes, actions, or behaviors (2). Goal-selection, goal-updating, and cognitive control can thus be considered central variables of the RDoC matrix for psychotherapy (**Table 1**). Psychotherapy can be divided into mechanisms, processes, and interventions that recreate cognitive control by establishing correspondence between goal selection and updating. Cognitive control has characteristic patterns in behavior and neurocircuitry and a frontal-cingulate-parietal-insular or “multiple demand” network forms a common functional substrate (38). Studies on neuropsychological performance show broad rather than distinct deficits in cognitive control across mental disorders, which correspond with aberrant activation and grey matter loss in the “multiple demand” network (38). The findings confirm the transdiagnostic and functional key significance of cognitive control. Due to the heritability of cognitive control capability, deficits have even been considered a risk factor or endophenotype of latent psychopathology vulnerability (38). If goals are not achieved, it is inevitably accompanied by a loss of control. Cognitive control must thus be supplemented by the RDoC construct of loss and sustained threat, which represent the complementary extreme value on the spectrum of need satisfaction (33). Loss and sustained threat encompass behaviors crucial for psychotherapy, such as avoidance, amotivation, or rumination (2). In contrast, the relationship of arousal/regulatory systems and other RDoC constructs with needs and psychotherapy may be far more complex, depending upon further variables or interacting across domains. It has to be taken into account that domain-specific processes partly correspond with disorder-specific mechanisms, as in, for example, hypervigilance, threat learning or avoidance learning with anxiety, and post-traumatic stress disorder, notwithstanding needs. This could limit the generalizability of a needs-based approach to any psychopathology (39, 40). Interactions among symptoms are investigated in recent translational models of psychopathology, such as the network approach, which assumes that mental disorders emerge from causal interactions among symptoms (41). However, network-based treatments have turned out not to be beneficial beyond the existing treatments, but require a large number of time series, repeated measurements, or cross-sectional data and are therefore methodologically complex (41). In process-based therapy, network models of psychopathology and change processes are analyzed to individualize treatment similar to a needs-oriented approach (10). Finally, the emotion regulation approach emphasizes the central importance of emotional dysregulation for all psychopathologies and highlights the simplification in diagnostics and treatment through this (42). Needs play a pivotal role in this approach because the change of emotional dysregulation requires awareness of desired goals and strategies to get there from one´s current state (42).

Obtaining basic needs or goals is rewarding and abnormal reward processing is a key feature of both addiction and transdiagnostic psychopathology (30, 43). Hence, a major intersection exists between basic needs in psychotherapy, on the one hand, and addiction research, on the other hand (44, 45). For example, an essential research question in addiction is how drugs shift and narrow the incentive away from the reward to the drug (44, 45). This probably involves the same neural mechanisms for reward processing and learning, which specify basic needs into individual goals. For addiction, these neurocognitive mechanisms have already been promoted in diagnosis, treatment, and clinical practice (46). Addiction research deals with the same content as psychotherapy from a different perspective. This could help to characterize and specify the processes of psychotherapy and further investigate the mechanisms found in empirical research to develop evidence-based interventions and treatments. Addiction research with paradigms such as the monetary incentive delay task could thus serve as a framework for the investigation of needs and the question of how basic needs neuronally specify into individual goals (**Table 1**; 30, 43).

Regardless of their theoretical background, all of the aforementioned concepts, such as principles of change in clinical psychology or consistency in neuropsychotherapy, have one similarity: they are all related to expectancies. For example, if an individual succeeds in prioritizing, joining, and balancing the urgency of needs with the availability of rewards and resources optimally, goals will be attained and a state of emotional balance, consistency, and mental health will occur. This requires and inevitably depends upon appropriate expectancies. In contrast, corrective experiences and missing problem awareness imply inappropriate expectancies and mental impairment. When needs and individual goals are at the center of treatment, they can more generally be categorized as expectancies. From this perspective, psychotherapy can be viewed as and simplified to addressing dysfunctional expectations (13). This understanding enables a mechanistic view of psychotherapy in terms of formal learning theory and cognitive neuroscience and confirms that needs and subjective preferences can be traced back to systematic processes and the underlying neural mechanisms of reward processing and learning (30, 45). Appropriate expectations require accurate learning from past experiences about their outcome on the basis of preceding cues or actions (13). In this sense, they are crucial for survival, and the evolutionary challenge is to arrive at adaptive expectations from only a limited set of contingency experiences (13). For this reason, erroneous expectations are both likely to arise and, at the same time, are a transdiagnostic feature of psychopathology. Examples are exaggerated fears in anxiety disorders and permanent pessimism in depression. In this view, dysfunctional expectations along with missing need satisfaction can be operationalized as reward prediction error in formal learning theory (13). Prediction errors rely on dopamine signaling in the mesolimbic pathway (13). Any rewarding stimuli such as food or drugs elicit activity of dopaminergic neurons within the ventral tegmental area and a subsequent release of dopamine in the nucleus accumbens (13). The dopamine activity corresponds with the mismatch between expected and received reward and with the omission of punishment, which is critical for fear extinction. Higher striatal dopamine activity is associated with better fear extinction learning and higher frontal dopamine activity with better fear extinction consolidation (13). As the dopaminergic coding of prediction errors in the mesolimbic pathway shapes learning, it is the foundation of adapting dysfunctional expectations and promoting corrective experiences in psychotherapy. Additionally, dopamine-based interventions can help to boost the effects of expectancy violation in psychotherapy (13). Pharmacologically, the administration of drugs that modulate phasic dopamine during exposure treatment could improve the acquisition of new safe memories and L-3,4-Dihydroxyphenylalanin (L-DOPA) administration after therapy reduces the return of fear in healthy individuals and improves consolidation (13). Behaviorally, working memory training increases activity in the prefrontal regions of the brain and cortical dopamine, as for example in obesity, resulting in increased response inhibition and retention of weight loss. All of these processes are of crucial importance to evaluate experiences accurately and thereby to arrive at appropriate expectations. Psychotherapy mainly occurs within the processes and underlying neural mechanisms of this spectrum between prediction errors and adaptive expectations, which comprises goal attainment and need satisfaction.

Addiction research supplies further evidence as to why psychotherapy takes place in this spectrum (47). Craving is an intense urge or desire to consume a drug and is a key feature of addiction (48). Substance-related cues involuntarily grab and hold attention and trigger approach behavior in individuals with addictive disorders (48, 49). At the same time, addiction is associated with impaired inhibitory control in the context of substance-related cues (50). These automatisms are the foundation of drug craving and they are rooted in subcortical brain regions beyond awareness (51). In psychotherapy, clients seek treatment when they are stuck in life because their usual habits do not enable them to cope with novel circumstances in their careers or relationships. Treatment thus aims at modifying unaware habits and automatisms, which are dysfunctional in a novel life situation, and makes the client aware of and allows them to adapt these habits (9). Automatic processes outside awareness have been assigned to the implicit system in dual-process theories of addiction (52). The implicit system narrows attention to drug cues and elicits a desire for, approach to, and use of drugs. In contrast, conscious control processes, such as impaired inhibition in drug addiction, are ascribed to the explicit system, which operates in parallel. Dual-process theories explain addiction as an imbalance between implicit automatic processes and explicit deliberate control processes (52). In this sense, addiction can be considered a malfunction of cognitive control, goal selection, and updating, and, similar to needs, can be ascribed to these RDoC variables (46). Dual-process models have been formulated for various mental disorders, such as depression, anxiety, and schizophrenia, and have been further applied to illustrate emotion regulation and transdiagnostically to investigate the neural mechanisms of change in psychotherapy treatment (15, 53, 54). Accordingly, implicit “bottom-up” emotion regulation features the absence of conscious supervision and explicit intention. Behavior is reinforced and automatized unconsciously based on rewarding and aversive outcomes and contingency (15). Thus, habits do not require voluntary attention and are automated, but goal-directed at the same time. Social norms such as the automatic shaking of hands and expectation of a friendly “hello” are examples of implicit emotion regulation (15, 55). Defense mechanisms in psychodynamic approaches or schemas in cognitive-behavioral therapy can be regarded as clinical forms of implicit emotion regulation (15, 55, 56). Schemas organize the individual´s appraisal of a situation automatically and unconsciously by framing it in similar experiences of the past and trigger former coping strategies (14, 56). These implicit emotional reactions can diverge from the requirements of a situation, distort feelings and thoughts, and interfere with conscious supervision, “top-down” intention, and explicit emotion regulation. The dual-process theory thus explains the cause of an emotional reaction, as it indicates an association between former experiences of a current situation and the potential interference of present requirements and intentions. As psychotherapy occurs on the spectrum between prediction errors and adaptive expectations, which encloses goal attainment and need satisfaction, explicit and implicit emotion regulation are probably the underlying processes operating in the background of this spectrum. The question is then how psychotherapeutic treatment adapts dysfunctional expectations and harmonizes emotion regulation, and in which neural mechanisms this adaptation is represented.

## Cognitive biases and reappraisal in treatment

A core principle of treatment in the large variety of psychotherapeutic approaches is restructuring or revision of semantic representations (**Figures 1a-c**) (15). Semantic representations are generalized regularities of experience, such as “dog bite”, whereby the regularities are neuronally disconnected from the related experiences. These appraisals arise out of individual emotional significance of daily experiences, consist of variable attributes such as, for example, “dog bite, smell, et cetera,” and incorporate interpersonal situations, the self, and others. They are a central constituent in models of emotional disorders in most psychotherapeutic approaches (15). The neurobiological correlates of semantic representations comprise the prefrontal cortex, the anterior temporal lobes, the temporo-parietal junction, and the inferior parietal lobe (15). This semantic system encodes the meaning of experience. The medial prefrontal cortex and the inferior parietal lobe are involved in emotional semantic representations (15). The anterior temporal lobes are associated with social cognition, conceptual knowledge of social behaviors, and representations of the self and social interaction. The anterior insula encodes rewarding or aversive regularities of the experience or the individual affective relevance.

Psychotherapy leads to mental recovery by improving cognitive control over emotion regulation (57). Dual-process models locate cognitive control in the prefrontal cortex (15). However, psychotherapy does not simply increase activation in the prefrontal cortex and thereby cognitive control. Rather, cognitive control is dependent on and determined by semantic representations. ^16^ For example, in a study, participants were requested to either remember or passively view faces. ^17^ Depending on the task, the prefrontal cortex modulated face-specific activity above or below the perceptual baseline. This top-down modulation was manifest in the fMRI activation magnitude of regions of the fusiform face area and in the processing speed in the N_170_ event-related potential. The prefrontal cortex thus enhances relevant information and suppresses irrelevant information for a task representation with this top-down mechanism (16). It allocates attention dynamically, restricts access to working memory, and activates representations in long-term memory in correspondence with a task (15). Cognitive control is generated by maintenance of activity patterns in the prefrontal cortex, which represent goals and the means to achieve them (16). They guide the flow of activity along neural pathways that establish the proper mappings between inputs, internal states, and outputs needed to perform a given task (16). In this way, any current semantic representation with associated goals and means blocks working memory, restricts perception, and determines cognitive control. A semantic representation is, thus, a bias in itself.

Present representations not only bias attention but also activate corresponding semantic representations in long-term memory (15, 16). For this reason, current representations with associated goals and means also restrict access to long-term memory and operate like a filter or bridge between working and long-term memory. Lewis-Peacock and Postle, for example, were able to retrieve the in working memory from the blood oxygenation level dependent signal of the previous long-term memory task (15, 16, 58). As semantic representations are generalized regularities out of individual experience, such as “*dog bite*”, it is plausible that the encounter with a dog activates corresponding representations in long-term memory. The main function of remembering, experience, and autobiographical memory is the faculty to draw on past experiences to plan and guide current behavior (18). If one´s only experience with dogs is a frightening and painful bite attack, it simply makes sense that any further encounter with a dog at first activates this specific experience with its associated notions, emotions, and behavior. This straightforward example of canophobia illustrates the association between experience, representation, anxiety, and flight as an automated behavioral reaction. It further emphasizes how specific representations inevitably elicit a discrete, predictable behavioral response and thus determine behavior (15). The foundation and functionality of representations can probably only be concluded from a phylogenetic or evolutionary perspective: the reliving of past events in the “here-and-now” enables us to draw on and create mental representations, which allow for error reduction, decision-making, planning, and finally goal-achievement (14, 59). Automated flight or behavioral reactions guarantee survival and fulfill a protective function. In contrast, dysfunction is the predisposition for mental disorders.

The experience with a dog potentially predicts the future approach to dogs and determines that subsequent events are encoded, recollected, and re-experienced in the same way. Current beliefs, or rather the person´s perspective on the event, shape autobiographical memory (18). Furthermore, the existing context, current goals, and motivation have an effect (18). For example, if one encounters the dog of the woman one has just fallen in love with, they are more likely to overcome their fear and approach her dog in order to be with her. Afterwards, emotional valence depends on goal attainment. Avoidance elicits negative emotions, reflects goal failure, and promotes detailed item-specific bottom-up processing for causal analysis, whereas an approach results in positive emotions, signals goal attainment, and thus generates heuristic, relational top-down processing to link present information with existing knowledge (18). The autobiographical memory of the *woman´s dog* is not rigid, but flexibly reconstructed in accordance with current goals and the personal perspective (18). At the same time, autobiographical memory maintains a coherent sense of self over time (18).

In other words, the individual emotional meaning of experience encoded in semantic representations determines a person. Psychotherapy creates consciousness of the appraisals, the related learning experiences, and behavioral habits. In reappraisal, the generalization of *“dog bite”* is traced to its learning experience and causes such as *“the dog was unleashed, injured, in pain and without a muzzle that day”* (15). Insight into the causes relativizes arousal and differentiates the appraisal. This enables the individual to derive coping strategies and finally gain back control over the experience. Cognitive reframing, as exemplified by the desire to approach *the woman*, creates a new context and perspective. This, in turn, increases motivation to overcome the fear of the dog (15, 60). A network in the prefrontal and orbitofrontal cortex stores experience of the affective value of encountered stimuli and situations that determine personal preferences (15). This part of the semantic system also encodes the prospective reward of actions taken in rewarding or aversive situations (15). It computes representations of subjective value in consideration of affective memories of past states and experiences, and present internal states such as drives or needs (15). With reappraisal, psychotherapy intervenes in this process. Ideally, reappraisal perfectly applies and adapts experiences to prospective goals and integrates them with needs. This comprises insight into the unaware contingency of automated behavior and in unaware experiences, which inevitably and involuntarily elicit associated behavior. It should also take goals, needs, and the current state of the person into account, for example, “*Am I in a condition to approach the woman, do I wish to be in a relationship, and is she a suitable partner?”* An optimal resolution of this process extends the restricted perspective of the personal meaning *“dog bite”* and the simple linkage *“run away!”* It bridges the self-containing biases in the neural processing of working memory, long-term memory, and autobiographical memory, and interrupts the feedback loop of re-experiencing the dog attack again. Reality testing and problem awareness promote the corrective experience *“the woman´s dog is friendly”*, which overwrites the former experience from the bottom up. Psychotherapy extends appraisal, makes automated behavior conscious, and fosters a functional behavioral reaction. Through this process, implicit emotion regulation aligns with explicit emotion regulation, establishing cognitive control over emotion regulation and self-regulation (**Table 1**). In this way, psychotherapy is a process that destroys dysfunctional expectations and promotes the accurate evaluation of experiences.

## Conclusion

In a basic theory of the mind, re-experiencing could be the most fundamental, evolutionarily justified functionality. Needs form the most appropriate central approach to treatment. Psychotherapeutic treatment can be regarded as the accurate evaluation of experiences with reappraisal interventions, which extend appraisal, make an individual aware of automated behavior, create a functional behavioral reaction, and target self-containing biases. Self-containing biases occur due to the processing of working and autobiographical memory. Cognitive control and working memory are RDoC constructs. Goal-attainment and need satisfaction comprise the RDoC subconstructs of goal and response selection and flexible updating. From an RDoC perspective on psychotherapy, needs are the most appropriate central approach, evaluation of experiences is the main mechanism, biases are the main target, and reappraisal is the main intervention. Psychotherapy ideally establishes cognitive control through goal attainment and need satisfaction. Diagnostically, it is difficult to find a distinguishing criterion for mental diseases on the level of needs. An application of needs in a clinical setting is the need-oriented therapeutic stance, which promotes positive social interaction in line with patients´ goals, in contrast to a pathologizing perspective. Addiction research could serve as a framework for the investigation of needs and the question of how needs are specified into individual goals. The investigation of neuronal mechanisms of individual characteristics could be a future direction for psychotherapy research. The challenges are implementation in clinical settings, the interdisciplinary exchange, and inconsistent terminology. Therefore, the mechanisms of the mind and the processes of psychotherapy can be operationalized as RDoC variables and the RDoC could serve as the foundation, framework, and needed guideline for the development of an evidence-based, universally-valid psychotherapy.
